# Disrupted SR–Mitochondria Coupling Drives Ischemia–Reperfusion Vulnerability in the Middle-Aged Rat Heart

**DOI:** 10.3390/biomedicines14030547

**Published:** 2026-02-27

**Authors:** Katarina Leskova Majdova, Maria Bencurova, Maria Kovalska, Peter Kaplan, Peter Racay, Zuzana Tatarkova

**Affiliations:** 1 Department of Medical Biochemistry, Jessenius Faculty of Medicine in Martin, Comenius University in Bratislava, 03601 Martin, Slovakia; majdova6@uniba.sk (K.L.M.); m.bencurova26@gmail.com (M.B.); peter.kaplan@uniba.sk (P.K.); peter.racay@uniba.sk (P.R.); 2 Department of Histology and Embryology, Jessenius Faculty of Medicine in Martin, Comenius University in Bratislava, 03601 Martin, Slovakia; maria.kovalska@uniba.sk

**Keywords:** myocardial ischemia–reperfusion, mitochondria-associated membranes, calcium homeostasis, redox signaling, lipid peroxidation

## Abstract

**Background**: Myocardial ischemia–reperfusion (IR) injury is associated with dysregulated Ca^2+^ handling and oxidative stress, particularly in the middle-aged heart. Sarcoplasmic reticulum (SR)–mitochondria communication via mitochondria-associated membranes (MAMs) is essential for coordinating Ca^2+^ transfer and redox signaling; however, its role in IR injury in the middle-aged myocardium remains incompletely understood. This study investigated changes in cardiac MAM protein composition and associated functional and oxidative parameters during ischemia and IR. **Methods**: Middle-aged rat hearts were subjected to global ischemia or IR using the Langendorff perfusion model. Mitochondrial, MAM, and homogenate fractions were analyzed using biochemical, proteomic, and functional assays to assess Ca^2+^-handling proteins, redox enzymes, lipid peroxidation markers, and mitochondrial antioxidant defenses. **Results**: Myocardial ischemia and IR disrupted SR–mitochondria communication in middle-aged hearts, leading to impaired Ca^2+^ handling, redox imbalance, and reduced contractile recovery. Ischemia induced significant MAM remodeling, characterized by reduced mitofusin 2 levels and increased enrichment of voltage-dependent anion channel 1. These changes were associated with disturbed mitochondrial Ca^2+^ signaling, impaired SR Ca^2+^ sequestration. Although mitochondrial antioxidant defenses, including MnSOD, were largely preserved, IR was associated with compartment-specific redox alterations within MAMs, as inferred from altered redox enzyme activity and enhanced lipid peroxidation. **Conclusions**: Disruption of SR–mitochondria coupling and MAM-associated redox regulation represents a key mechanism underlying increased vulnerability to IR injury in the middle-aged heart. Targeting MAM integrity and modulating Ca^2+^-redox cross-talk may improve cardiac resilience in elderly populations.

## 1. Introduction

According to the World Health Organization, cardiovascular diseases remain the leading cause of death globally, with recent estimates indicating nearly 20 million deaths per year. The heart is the most metabolically active organ, containing the highest density of mitochondria, which occupy more than 30% of the cardiomyocyte volume and are located close to the main energy-consuming sites—the myofilaments, sarcoplasmic reticulum (SR), and T-tubules [1]. Numerous studies have reported that cardiomyocyte pathophysiology is associated with alterations in mitochondrial morphology (e.g., structural deformation, swelling, loss, or reorientation of cristae) as well as impaired physiological function of the heart [2,3], such as the development of arrhythmias [4]. Beyond their central role in energy production, mitochondria also participate in amino acid interconversion, β-oxidation of fatty acids, and the synthesis of heme and Fe–S clusters [3]. Another multifunctional organelle, the endoplasmic reticulum (ER), plays a crucial role in protein folding and processing [5], lipid biosynthesis, calcium storage, and the formation of autophagosomes and peroxisomes [5,6]. Conversely, dysfunction of these organelles is associated with disrupted intracellular Ca^2+^ homeostasis [7,8], increased reactive oxygen species production [9], reduced mitochondrial energy generation [10], and ER stress–induced impairment of protein folding, which carries the risk of proteotoxicity [11,12].

The sarcoplasmic reticulum in cardiac muscle serves as a high-capacity Ca^2+^ reservoir essential for regulating contraction and relaxation [11]. Its function is tightly linked to mitochondrial Ca^2+^ homeostasis, and disturbances in this cross-talk are closely associated with cardiac remodeling [4,13]. Mitochondria act in synergy with the SR and other organelles to regulate both intra- and extracellular microenvironments, particularly Ca^2+^ dynamics, signaling pathways, metabolism, and intercellular communication [14]. Although mitochondria and the SR have distinct roles, they complement one another through specialized contact sites known as mitochondria-associated membranes (MAMs) [15]. Over the past decade, the molecular structure and function of more than 1000 MAM-associated proteins have been identified [16]. However, the density of MAM connections, their structural organization, and their functional roles vary substantially across species, organs, and cell types [17,18]. A major challenge remains the comprehensive profiling of MAMs-localized proteome in different cell types [19]. This requires highly purified MAM fractions and the ability to distinguish resident proteins (core or auxiliary) from transient proteins that appear only during dynamic contact events—factors that may critically influence mitochondria–SR communication.

The transfer of Ca^2+^ from the endoplasmic/sarcoplasmic reticulum to mitochondria is mediated by the main MAM protein complex, which consists of the voltage-dependent anion channel 1 (VDAC1) located on the outer mitochondrial membrane and the inositol 1,4,5-trisphosphate receptor (IP_3_R) on the ER membrane, both stabilized by glucose-regulated protein 75 (GRP75) [20]. VDAC1 facilitates mitochondrial Ca^2+^ influx by coupling with Ca^2+^ release through IP_3_R channels in non-excitable cells [21] or through ryanodine receptors (RyR) in excitable cells [22]. In cardiac muscle, mitochondria-produced ATP further stimulates the rapid release of SR-derived Ca^2+^ into the cytoplasm, where it binds to troponin and triggers myocardial contraction [23]. However, excessive mitochondrial Ca^2+^ uptake can initiate stress responses and impair mitochondrial function.

Over recent decades, most animal studies have relied on young adult rodents, which display high regenerative capacity compared with older animals. Conversely, senescent hearts demonstrate greater stress tolerance [24]. Based on these considerations, we selected middle-aged animals as a model to investigate the effects of acute stress induced by ischemia and/or ischemia–reperfusion. Our study specifically focused on Ca^2+^ homeostasis, the extent of oxidative damage, and adaptive capacity.

## 2. Materials and Methods

### 2.1. Animals

A total of 30 male Wistar rats, aged 14 months, were obtained from the Institute of Experimental Pharmacology, Slovak Academy of Sciences (Dobra Voda, Slovakia). The animals were maintained on a 12 h light/dark cycle with free access to standard rodent chow and water in a temperature-controlled room (22 ± 2 °C). All procedures were conducted following the EU Directive 2010/63/EU on the protection of animals used for scientific purposes and were approved by the Ethical Committee of the Jessenius Faculty of Medicine in Martin and the State Veterinary and Food Department of Slovakia (No. 554/11-221/3). Animals were randomly assigned to three experimental groups, and their hearts were subjected to the conditions described below. After the induction of ischemia–reperfusion injury, the group allocation was concealed using coded identifiers to ensure blinded analysis.

### 2.2. Langendorff’s Model of Global Ischemia and Reperfusion

Animals were euthanized following deep anesthesia. Anesthesia was induced using halothane (3% in an O_2_/N_2_O mixture, 1:2). After confirmation of deep anesthesia (absence of reflexes), the animals were decapitated, and the hearts were excised. The hearts were immediately placed in ice-cold Krebs–Henseleit (K-H) solution (135.0 mM NaCl, 5.4 mM KCl, 0.9 mM MgCl_2_, 24 mM NaHCO_3_, 1.2 mM NaH_2_PO_4_, 1.8 mM CaCl_2_, 10 mM glucose; pH 7.4). Hearts were cannulated via the aorta and perfused at 37 °C under a constant pressure of 73 mmHg using the Langendorff system (ML870B2, ADInstruments, Spechbach, Germany). The K-H solution was continuously bubbled with a gas mixture of 95% O_2_/5% CO_2_. Left ventricular pressure (LVP) was measured using a latex balloon inserted into the left ventricle via the left atrium and connected to a pressure transducer. The balloon volume was adjusted to maintain an initial left ventricular end-diastolic pressure of 8–12 mmHg. Temperature and hemodynamic parameters were continuously monitored using the PowerLab 8/30 Data Acquisition System with Chart Software (ML870B2, ADInstruments, Spechbach, Germany).

Following a 15 min equilibration period, hearts were subjected either to 20 min of global no-flow ischemia (ISCH) or to 20 min of ischemia followed by 30 min of reperfusion (IR). Control hearts were perfused for a total of 65 min (CON). The Langendorff model with a 20 min no-flow ischemia was chosen because this ischemic period has been reported to induce minimal cardiomyocyte necrosis while allowing full recovery of post-ischemic function [25]. At the end of the protocol, all hearts were snap-frozen and stored at −70 °C until used for the preparation of homogenates and the isolation of mitochondria, SR, and MAM fractions.

### 2.3. Isolation of Subcellular Fractions and Protein Concentration Assay

Tissue homogenates were prepared from 14-month-old rats (*n* = 30). Frozen tissues were thawed in 10 volumes of ice-cold homogenization buffer (30 mM KH_2_PO_4_, 5 mM EDTA, 0.3 M sucrose; pH 7.0) containing 0.3 mM phenylmethylsulfonyl fluoride as a protease inhibitor, and homogenized five times at 1000 rpm using 25 s/20 s intervals with a precooled Teflon pestle in a Potter–Elvehjem homogenizer. Half of the homogenates (*n* = 15; 5 per group) were used for the isolation of SR vesicles as previously described in detail [26,27]. The remaining homogenates (*n* = 15; 5 per group) were used for isolation of mitochondria and MAM fraction following a slightly modified method [28]. Briefly, the mitochondria-MAM mixed fraction was obtained by differential centrifugation (3200× *g*, 10 min), and the resulting supernatant was further centrifuged at 10,000× *g* for 40 min using Optima L-100 XP ultracentrifuge (Beckman Coulter, Brea, CA, USA). The resulting mitochondria-MAM fraction was resuspended in 2 mL of ice-cold MRB solution (250 mM mannitol, 5.0 mM HEPES, 0.5 mM EGTA; pH 7.4).

For gradient ultracentrifugation, Percoll medium (225 mM mannitol, 25 mM HEPES, 1 mM EGTA; pH 7.4, supplemented with fresh 30% Percoll) was used. The mixture was layered as follows in an ultracentrifuge tube: 8 mL of Percoll medium, 2 mL of the mitochondria-MAM fraction, and MRB solution to fill the tube up to 0.5 cm below the top. Ultracentrifugation was performed at 95,000× *g* for 30 min on Optima L-100 XP ultracentrifuge (Beckman Coulter, Brea, CA, USA). The upper MAM ring and lower mitochondrial ring were collected separately and centrifuged at 6300× *g* for 10 min. The mitochondrial pellet was resuspended in 0.5 mL MRB solution. The MAM-containing supernatant was further centrifuged at 100,000× *g* for 1 h, and the resulting MAM pellet was dissolved in 0.2 mL MRB solution. All isolation steps were performed at 4 °C, and the final fractions were stored at −70 °C for subsequent experiments. To assess the relative purity of mitochondrial and MAM fractions, VDAC distribution was analyzed by Western blot as mentioned in Section 2.7. The highest enrichment was observed in purified mitochondria, with intermediate levels in MAM and lower levels in crude mitochondria, supporting successful fractionation. Representative blots are shown in Supplementary Figure S1.

The total protein concentration was determined using the DC Protein Assay Kit (500-0111, Bio-Rad Laboratories, Hercules, CA, USA) with bovine serum albumin as the standard, following the manufacturer’s instructions. Absorbance was measured with a BioTek Synergy H4 Hybrid Microplate Reader (Agilent Technologies, Santa Clara, CA, USA). All results are summarized in Table A1.

### 2.4. Assay of Ca^2+^-ATPase Activity and Kinetic Parameters

Cardiac sarco-endoplasmic reticulum Ca^2+^-ATPase (SERCA) activity was determined according to the method [26] using a PharmaSpec UV-1700 spectrophotometer (Shimadzu, Kyoto, Japan). Mg^2+^-dependent, Ca^2+^-stimulated ATPase activity was assessed by measuring the rate of inorganic phosphate release.

The reaction medium contained 20 mM HEPES (pH 7.0), 100 mM KCl, 5 mM MgCl_2_, 5 mM sodium azide, 5 mM ATP, 5 μg/mL Ca^2+^ ionophore A23187, 0.1 mM EGTA, variable volumes of 1 mM CaCl_2_ corresponding to free Ca^2+^ concentrations of 0.02–5.00 μM, and 0.05 mg/mL SR proteins. Reactions were initiated by the addition of ATP and terminated after 15 min of incubation at 37 °C by adding 10% trichloroacetic acid on ice (4 °C). SERCA activity was calculated as the difference between total Mg^2+^, Ca^2+^-ATPase activity, and Mg^2+^-ATPase activity measured in the absence of CaCl_2_ (in the presence of 3 mM EGTA). Kinetic parameters were determined using nonlinear least-squares fitting, according to the equation: V_max_/v = 1+(K_Ca_/[Ca^2+^]), where v is the reaction velocity, V_max_ is the maximal activity of SERCA, [Ca^2+^] is the free Ca^2+^ concentration, and K_Ca_ is [Ca^2+^] at V_max_/2.

### 2.5. Assay of Antioxidant Enzymes Activity and Glutathione Content

Cardiac homogenates were used to assay glutathione reductase (GR), glutathione peroxidase (GPx), and thioredoxin reductase (TrxR) activities. Enzyme activities were determined according to the manufacturer’s protocols using commercially available kits: GR Assay Kit (GRSA, Sigma-Aldrich, Munich, Germany), GPx Cellular Activity Assay Kit (CGP1, Sigma-Aldrich, Munich, Germany), and TrxR Assay Kit (10007892, Cayman Chemical, Ann Arbor, MI, USA). Measurements were performed with a BioTek Synergy H4 Hybrid Microplate Reader (Agilent Technologies, Santa Clara, CA, USA).

Manganese superoxide dismutase (MnSOD) activity was determined based on the inhibition of nitroblue tetrazolium (NBT) reduction by superoxide generated from xanthine oxidase. The reaction mixture contained 0.05 mM phosphate buffer (pH 7.8), 0.1 mM EDTA, 0.025 mM NBT, and 0.1 mM xanthine. Absorbance changes were recorded at 550 nm against a control using a PharmaSpec UV-1700 spectrophotometer (Shimadzu, Kyoto, Japan). One unit of enzyme activity was defined as the amount of enzyme required to cause 50% inhibition of NBT reduction [29].

The concentration of reduced glutathione (GSH) in cardiac homogenates was determined using a Glutathione Assay Kit (354102, Calbiochem, San Diego, CA, USA), following the manufacturer’s instructions. Briefly, 0.02 mL of the sample and 0.05 mL of chromogen in HCl solution were added to the phosphate buffer. After the addition of 30% NaOH, the reaction mixture was incubated for 10 min in the dark. Absorbance was measured at 400 nm against GSH standards, and results were expressed as μmol/g of heart tissue.

### 2.6. Evaluation of Lipid Peroxidation and Membrane Hydrophobicity

The formation of conjugated dienes was estimated from the absorbance ratio A_233_/A_215_ in mitochondrial samples (0.02 mg/mL) suspended in 10 mM phosphate buffer containing 1% Lubrol. The level of 4-hydroxy-2-nonenal (4-HNE), a secondary product of lipid peroxidation (LPO), was determined in cardiac tissue homogenates using the Bioxytech HAE-586 Assay Kit (21043, OxisResearch, Portland, OR, USA). Butylated hydroxytoluene (0.5 M) was added during homogenization to prevent sample oxidation.

Fluorescence measurements with the 1-anilino-8-naphthalenesulfonate (ANS) probe were performed at 25 °C in buffer containing 10 mM HEPES and 100 mM KCl (pH 7.0) with 0.05 mg of mitochondrial protein. Probe binding was assessed after 10 min incubation by measuring fluorescence emission at 480 nm (slit width 5 nm) following excitation at 365 nm (slit width 5 nm). Emission spectra (425–480 nm) of lysine conjugates with LPO-derived products (Lys–LPO) were recorded after excitation at 365 nm (slit width 5 nm) using an LS-55 spectrofluorometer (PerkinElmer, Waltham, MA, USA), as previously described [26]. Results are expressed as fluorescence intensity in arbitrary units (AU).

### 2.7. Western Blot and Immunodetection

For detection of proteins (VDAC1, GRP75, MFN2, SOD2, Cyt c, SERCA2), tissue homogenates (0.03 mg) or mitochondrial/MAM protein samples (0.02 mg) were separated by sodium dodecyl sulfate-polyacrylamide gel electrophoresis (SDS-PAGE) and transferred onto nitrocellulose membranes using a Trans-Blot^®^ SD Semi-Dry Transfer Cell (1620115, Bio-Rad Laboratories, Hercules, CA, USA). To assess total protein loading control, membranes were stained with Ponceau S for total protein visualization for 5 min. Non-specific binding was blocked overnight in TBST (Tris-buffered saline containing 0.05% Tween-20) supplemented with 5% non-fat dry milk. The following day, membranes were incubated with primary antibodies (Santa Cruz Biotechnology, Dallas, TX, USA): polyclonal rabbit anti-MFN2 (sc-50331, 1:200) and goat anti-actin (sc-1616, 1:1000), monoclonal anti-GRP75 (sc-133137, 1:200), anti-VDAC1 (sc-390996, 1:500), anti-SOD2 (sc-133134, 1:1000), anti-Cyt c (sc-13560, 1:200) and SERCA2 (sc-53010, 1:500).

After washing with TBST, membranes were incubated for 1 h with appropriate secondary antibodies. For monoclonal primary antibodies, goat anti-mouse IgG–HRP (sc-2005, 1:1000; Santa Cruz Biotechnology, Dallas, TX, USA) was used. Polyclonal antibodies were detected using biotinylated secondary antibodies (Agilent Technologies, Santa Clara, CA, USA): rabbit anti-goat IgG (Dako, E0466; 1:10,000) for actin and goat anti-rabbit IgG (Dako, E0432; 1:10,000) for MFN2, applied for 1 h. Following washing, membranes were incubated for 30 min with an ABC Peroxidase Staining Kit (32020, Thermo Fisher Scientific, Waltham, MA, USA), followed by thorough washing in TBST. Immunoreactive proteins were visualized after 5 min exposure to SuperSignal™ West Pico Chemiluminescent Substrate (34080, Thermo Fisher Scientific, Waltham, MA, USA) in the dark, using a ChemiDoc™ XRS Imaging System. Band intensities were quantified with Quantity One software v5.0 (both Bio-Rad Laboratories, Hercules, CA, USA). Results are expressed as relative protein levels in arbitrary units (AU).

### 2.8. Histomorphological Analysis

Hearts removed from the Langendorff perfusion system were fixed in 4% paraformaldehyde for 24 h, followed by immersion in 30% sucrose for an additional 24 h. The tissue was then frozen and sectioned using a cryostat (Thermo Fisher Scientific, Waltham, MA, USA). Serial coronal heart sections (30 μm thick) were obtained from three animals per group, with every third section collected over an approximately 3 mm span. Sections were stained with hematoxylin and eosin (H&E). Histopathological evaluation was performed by two independent investigators blinded to group allocation. Five sections per animal were analyzed, and three randomly selected fields from different regions of the left ventricle (LV) were examined per section. Slides were scanned at 200× magnification using a VisionTek digital scanning microscope (Sakura Finetek USA, Torrance, CA, USA). Svslide images were analyzed with VisionTekViewer 2.6 software (Sakura Finetek USA, Torrance, CA, USA). Particle analysis was conducted with size thresholds set from 0 mm^2^ to infinity without shape restrictions. The observation frame was defined as 1 mm^2^ (1 × 1 mm). Histopathological changes were quantified using manual assessment with software-assisted analysis.

### 2.9. Statistical Data Analysis

Statistical analyses were performed using GraphPad InStat, version 3.01 (GraphPad Software, San Diego, CA, USA). Data are presented as mean ± standard error of the mean (SEM). Differences between experimental groups were evaluated by one-way analysis of variance (ANOVA) followed by the Student–Newman–Keuls post hoc test. A *p*-value < 0.05 was considered statistically significant.

## 3. Results

### 3.1. Effect of IR on Contractile Function Parameters

The effects of ischemia–reperfusion (IR) on contractile function in middle-aged hearts are summarized in Table 1. To minimize variability among control values, all parameters were recorded at identical time points; therefore, the control group included both continuously perfused hearts and the 15 min stabilization period of IR hearts prior to ischemia. Myocardial IR injury resulted in a significant reduction in coronary flow, which decreased to approximately 88% of control values, whereas heart rate remained unchanged. Developed left ventricular pressure (LV devP) declined by 20.7% (*p* < 0.01), from a pre-ischemic value of 87.6 ± 2.7 mmHg to 69.5 ± 3.1 mmHg after 30 min of reperfusion. Overall, ventricular contractile performance was significantly impaired following IR. Specifically, the maximum rate of pressure development (+LV dP/dt) was reduced by 35.5%, decreasing from a pre-ischemic value of 1591.5 mmHg/s to 1026.2 mmHg/s, indicating a diminished capacity of 14-month-old hearts to recover systolic function. In contrast, the maximum rate of relaxation (−LV dP/dt) was relatively preserved, maintaining 73.2% of its pre-ischemic value.

### 3.2. Hematoxylin and Eosin Staining

**Figure 1:**
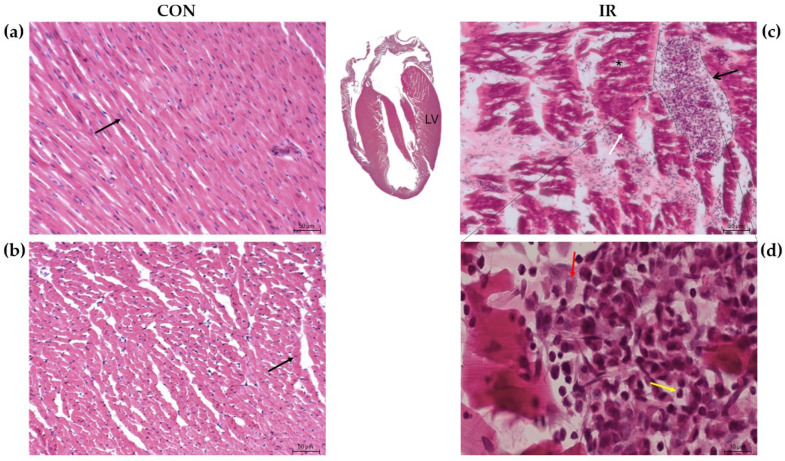
Representative Hematoxylin and Eosin (H&E)-stained microphotographs of rat myocardium from control (CON) and ischemia–reperfusion (IR) groups (*n* = 3 per each experimental group). Control myocardium is shown in longitudinal (**a**) and cross-sectional (**b**) views (scale bar = 50 μm). IR myocardium is shown at 200× (**c**) and 630× (**d**) magnification. Arrows indicate normal nuclei (black), granular tissue (open black), intensely eosinophilic cardiomyocytes (white), macrophages (red), and neutrophils (yellow); asterisks indicate early edema. LV (left ventricle).

Based on the analysis of physiological parameters, we focused on the left heart for routine histological evaluation. The left ventricle from each animal was collected for analysis. H&E staining revealed no pathological alterations in the control group. Cardiomyocytes appeared as polygonal cells with a cylindrical morphology and a centrally located nucleus. The perinuclear cytoplasm was devoid of myofibrils but enriched with organelles. Cardiomyocytes were orderly arranged, uniformly stained with no apparent signs of cell degradation, necrosis, or inflammatory infiltration (Figure 1a,b). On the other hand, the IR group (Figure 1c,d) showed diffuse areas with more eosinophilic, wavy-appearing cardiomyocytes, early signs of interstitial edema (separation of fibers due to fluid accumulation), and, rarely, areas of infiltration by macrophages and neutrophils.

Since the ischemia–reperfusion period was short and designed to assess acute myocardial injury, it was insufficient to induce detectable collagen deposition or fibrotic remodeling, which typically occur at later stages. In addition, potential age-related baseline collagen deposition could confound interpretation. Therefore, Masson’s trichrome staining was not performed, and H&E staining was considered adequate for evaluating the observed histomorphological changes.

### 3.3. Effect of IR on Ca^2+^-ATPase Activity

**Figure 2:**
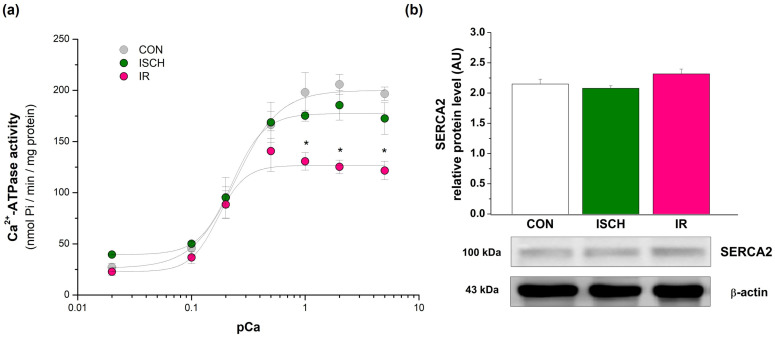
Calcium dependence of Ca^2+^-ATPase activity (**a**) after induced ischemia and reperfusion and representative Western blot for relative SERCA2 protein level (**b**). The nonlinear least-squares method with Hill fitting was used for the graphical interpretation of Ca^2+^ dependence. The values are expressed as mean ± SEM of 5 hearts per experimental group. A one-way ANOVA with post hoc comparisons by the Student–Neuman–Keuls test was carried out to test for significant differences (*) when compared to the control: * *p* < 0.05.

Myocardial ischemia–reperfusion significantly (*p* < 0.05) impaired Ca^2+^-ATPase activity at higher free Ca^2+^ concentrations (1–5 μM) compared with control hearts and hearts subjected to 20 min of global ischemia. The affinity of Ca^2+^-ATPase for free Ca^2+^, assessed at concentrations up to the half-maximally activating Ca^2+^ concentration, was similar between the CON and ISCH groups but was reduced in the IR group (Figure 2a). Moreover, the Hill coefficient was markedly increased in the IR group (3.62), indicating altered Ca^2+^ binding and reduced Ca^2+^ sensitivity. These findings suggest impaired Ca^2+^-sequestering capacity in hearts subjected to acute ischemia–reperfusion. Western blot analysis revealed no significant differences in SERCA2 protein expression among CON, ISCH, and IR groups. Densitometric quantification normalized to the respective loading control confirmed comparable SERCA2 levels across all experimental conditions (Figure 2b).

### 3.4. Western Blot and Immunodetection

**Figure 3:**
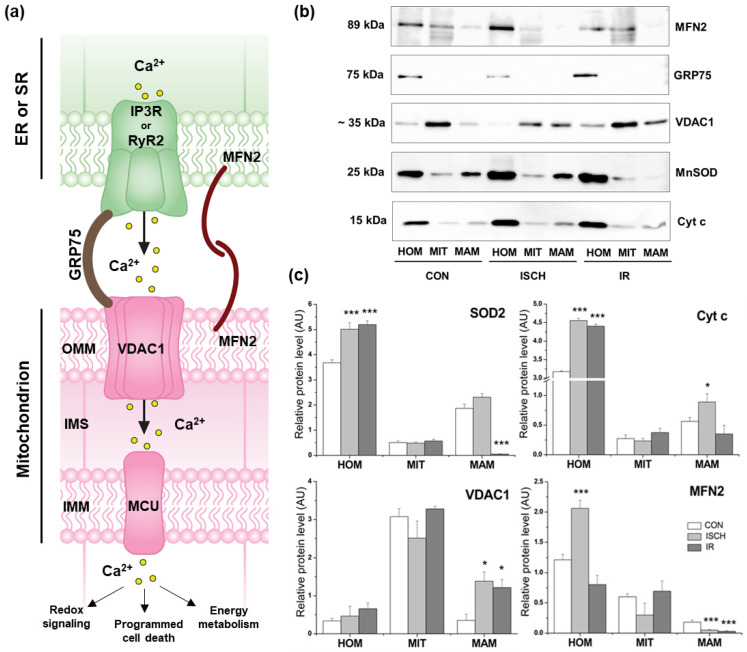
Mitochondria–ER/SR coupling in Ca^2+^ homeostasis after induced ischemia and reperfusion. Scheme of ER/SR–mitochondria communication (**a**) with Western blots (**b**) and quantitative evaluation of relative protein contents (**c**) in comparison to the loading control. The values are expressed as mean ± SEM of 3 hearts per experimental group. A one-way ANOVA with post hoc comparisons by the Student–Neuman–Keuls test was carried out to test for significant differences (*) when compared to the control: * *p* < 0.05, *** *p* < 0.001. Cyt c (cytochrome c), ER (endoplasmic reticulum), GRP75 (glucose-regulated protein 75), HOM (homogenate), IMS (intermembrane space), IP3R (inositol 1;4;5-trisphosphate receptor), MAM (mitochondria-associated membrane), MIT (mitochondria), MFN 2 (mitofusin 2), MnSOD (Mn superoxide dismutase), MCU(mitochondrial Ca^2+^ uniporter), OMM and IMM (outer and inner mitochondrial membrane), RyR2 (ryanodine receptor 2), SR (sarcoplasmic reticulum), VDAC1 (voltage-dependent anion channel 1).

The main Ca^2+^-transfer regulatory contact sites were assessed in tissue homogenates, mitochondria, and MAMs simultaneously. Ischemia induced a decrease in mitochondrial VDAC1 levels, accompanied by a significant 3.9-fold increase in its content within the MAM fraction. This was paralleled by elevated cytochrome c levels in both homogenates and MAMs from ISCH hearts (Figure 3b,c). Upregulation of VDAC1, through interactions with pro-apoptotic proteins, may facilitate cytochrome c release from mitochondria into the cytosol, as observed after IR. Although various stressors such as glucose deprivation, oxidative stress, and hypoxia are known to induce GRP75, 20 min of ischemia resulted instead in a reduction in GRP75 content in homogenates (Figure 3b). GRP75 was undetectable in mitochondria or MAMs; thus, no statistical analysis is presented. This may reflect partial loss during fractionation, redistribution to the cytosol, where substantial cytosolic pools of GRP75 have been reported, or levels below the detection limit. Another mitochondria-specific protein, MFN2, which contributes to SR–mitochondria tethering, was also affected. Ischemia reduced mitochondrial MFN2 content to 50% of control levels, with partial recovery after IR, whereas MFN2 in the MAM fraction declined to 27.5% after ISCH and was further decreased during reperfusion (Figure 3b,c).

### 3.5. Effect of Ischemia–Reperfusion on Antioxidant Molecules

To assess oxidative stress, several antioxidant parameters were measured in 14-month-old rat hearts following ischemia (ISCH) and ischemia–reperfusion (IR). Reduced glutathione (GSH) levels did not differ between the control and IR groups (Table 2); however, ischemia alone decreased GSH content to 64.2% of control (*p* < 0.001). Enzymes involved in GSH metabolism, including glutathione peroxidase and glutathione reductase, were unchanged, whereas thioredoxin reductase activity increased significantly after ischemia, from 31.38 ± 1.82 to 45.33 ± 1.25 nmol/(min·mg protein). Mitochondrial MnSOD activity and protein levels (Figure 3c) were unaffected by ISCH or IR, although high MnSOD content was detected in the MAM fraction in both control and ischemic groups.

### 3.6. The Extent of Membrane Lipid Peroxidation and Hydrophobicity

To evaluate the impact of ischemia–reperfusion on membrane lipids, several markers of lipid peroxidation were assessed. As shown in Table 3, conjugated diene formation increased by 69.7% (*p* < 0.001) in the ischemia (ISCH) group and by 44.9% in the IR group. These changes were accompanied by a significant increase in the lipid peroxidation product 4-hydroxy-2-nonenal (HNE), from 12.57 ± 1.32 to 21.82 ± 1.22 nmol/g in ischemic hearts, whereas no increase was observed after IR. In contrast, binding of lipid peroxidation–derived products to lysine residues did not differ among the three experimental groups. Increased lipid peroxidation was further reflected by a significant increase in ANS probe binding (*p* < 0.001) to membrane proteins, indicating structural alterations of proteins and/or membrane surfaces.

## 4. Discussion

SR–mitochondria coupling belongs to a group of key regulators of myocardial metabolism [30]. While SR-Ca^2+^ is essential for the EC coupling and regulates Ca^2+^ release to the cytosol in cardiomyocytes [31], the Ca^2+^ uptake by mitochondria is a regulator of ATP production required for muscle contraction. The contact sites between SR and mitochondria cover 2–5% of the mitochondrial surface to perform various functions between both organelles, such as lipid exchange and Ca^2+^ transfer, apoptosis, mitochondrial biogenesis, and dynamics [17,22,32,33].

The opening of the sarcoplasmic reticulum (SR)–resident Ca^2+^ release channel RyR in excitable cells activates Ca^2+^ influx into mitochondria through VDAC1 (voltage-dependent anion channel 1) located on the outer mitochondrial membrane (OMM) [22]; however, excessive Ca^2+^ uptake may initiate cellular stress events. GRP75, as a modulator of the IP_3_R–VDAC1 complex [34,35], increases the efficiency of mitochondrial Ca^2+^ uptake [36], although its overexpression does not affect the number of contact sites. In contrast, inhibition of GRP75 decreases mitochondrial permeability transition pore opening by regulating the interaction of cyclophilin D with this complex, thereby protecting cardiomyocytes from lethal hypoxia–reoxygenation injury [37]. In the present study, we detected a potentially protective decrease in GRP75 levels after 20 min of global ischemia. Similarly, complete depletion of GRP75 was previously shown to confer protection against stress [38] and to maintain mitochondrial homeostasis [39]. Increasing experimental evidence suggests that GRP75-linked SR–mitochondria coupling is an important determinant of cell fate under stress conditions without directly affecting mitochondrial function. In this context, GRP75 has been identified as a critical mediator of ER/SR–mitochondria Ca^2+^ transfer and cardiomyocyte calcium homeostasis during ischemia–reperfusion injury [40]. Moreover, substantial cytosolic pools of GRP75 have been reported [41,42], which may complicate precise subcellular localization and interpretation of fractionation-based analyses under stress conditions.

VDAC1, which is located near the SR within mitochondria-associated membranes (MAMs) [43], functions in concert with adenine nucleotide translocase and mitochondrial creatine kinase. In cardiomyocytes, VDAC1 transfers apoptotic Ca^2+^ signals into mitochondria [44], leading either to OMM permeabilization or to protective responses. We observed elevated VDAC1 content in the MAM fraction after ISCH and IR, which may reflect an adaptive attempt to preserve cardiomyocyte function. Conversely, VDAC1 overexpression may promote its interaction with pro-apoptotic proteins, facilitating cytochrome c release from mitochondria [45]. In our experiments, increased cytochrome c content in MAMs was detected only after ISCH, potentially rendering cardiomyocytes more vulnerable to pro-apoptotic or stress signals.

Mitofusin 2 (MFN2) is critical for coupling cardiac contractile activity with metabolism, most likely by maintaining close spatial proximity between the SR and mitochondria within MAMs to optimize Ca^2+^ transfer [46]. This tethering represents strict physical interaction, independent of GTPase activity, and is primarily formed between the small (~10%) fraction of MFN2 localized in the SR membrane and OMM-associated MFN2 [47]. In the present study, ischemia induced a reduction in mitochondrial MFN2 protein levels, accompanied by a more pronounced decrease in MFN2 content within the MAM fraction. MFN2 performs localization-dependent functions. At the outer mitochondrial membrane, it regulates mitochondrial fusion, which may support mitochondrial integrity under stress conditions and could explain its partial preservation in total mitochondrial fractions. In contrast, MAM-associated MFN2 modulates ER/SR–mitochondria tethering and Ca^2+^ transfer. The preferential depletion of MFN2 from MAMs therefore suggests selective remodeling of membrane contact sites during ischemia. Reduced MFN2 expression has previously been observed in failing guinea pig cardiomyocytes [48] despite preserved mitochondrial membrane potential, suggesting that MFN2 loss does not directly impair ATP production [49]. However, MFN2 deficiency mediates mitochondrial morphological alterations, reduces close contact sites [50], and has been associated with increased ROS production and impaired Ca^2+^ handling [49]. Notably, MFN2 has been proposed to act not merely as a structural tether but also as a regulator of ER–mitochondria coupling. Accordingly, its downregulation during stress may alter Ca^2+^ homeostasis and inter-organelle communication [40,51]. While MFN2 ablation has been reported to enhance SR-to-mitochondria Ca^2+^ transfer efficiency [47] and reduce sensitivity to IR injury [52], other studies have shown that MFN2 removal increases SR–mitochondria distance, thereby reducing mitochondrial Ca^2+^ uptake [53] and disrupting normal SR coupling [54]. Under such conditions, Ca^2+^ may be preferentially reabsorbed by the SR, resulting in a gradual increase in SR Ca^2+^ stores. Collectively, these findings suggest that differential MFN2 depletion reflects dynamic and compartment-specific remodeling of SR–mitochondria communication during ischemia, potentially balancing mitochondrial quality control with adaptive modulation of Ca^2+^ signaling.

Emerging evidence suggests that SR–mitochondria coupling and MAM remodeling are not regulated solely by local protein interactions but also by upstream transcriptional programs. In particular, serum response factor (SRF), a key regulator of cardiac growth and stress adaptation, has recently been implicated in mitochondrial regulation during cardiac aging. Recent studies [55,56] demonstrated that altered SRF signaling affects mitochondrial bioenergetics, redox balance, and structural integrity in aging myocardium. Given that aging is associated with impaired inter-organelle communication, SRF-dependent transcriptional reprogramming may contribute to remodeling of MAM-associated proteins and altered SR–mitochondria coupling during ischemia–reperfusion. Although not directly examined here, this mechanism provides a plausible upstream link between cardiac aging and the structural and functional changes observed in our study.

In cardiomyocytes, Ca^2+^-ATPase (SERCA) serves as the primary SR Ca^2+^ loading system [57]. During diastole, approximately 15% of cytosolic Ca^2+^ is transiently taken up by mitochondria, whereas the majority is handled by the cardiac SERCA2a isoform [58] and the Na^+^/Ca^2+^ exchanger [59,60]. Downregulation of SERCA2a expression and activity has been reported in various cardiac pathologies [26,61,62], often resulting in an increased phospholamban (PLN)-to-SERCA2a ratio [27] which is consistent with impaired SR Ca^2+^ uptake. In the present study, the observed decrease in Ca^2+^-ATPase activity was not accompanied by decreased SERCA2 protein abundance, suggesting that functional decline might be related to the ROS-mediated post-translational modifications of SERCA2 protein. Although oxidative modifications were not directly assessed here, previous studies have demonstrated that SERCA2a activity is inhibited during myocardial ischemia–reperfusion (IR) through oxidation of cysteine residues and tyrosine nitration [63,64]. Collectively, these findings suggest that middle-aged hearts subjected to IR exhibit a limited Ca^2+^ transport capacity, leading to cytosolic Ca^2+^ accumulation. This observation is in line with prior reports describing Ca^2+^ overload and ROS-mediated cardiomyocyte death under IR conditions [58,65]. Additionally, a thioredoxin-related transmembrane protein localized in MAMs [66] can inhibit Ca^2+^-ATPase via enhanced mitochondria-derived ROS production and SR Ca^2+^ dissipation [67], thereby further contributing to intracellular Ca^2+^ accumulation.

Multiple factors modulate SR–mitochondria interactions, among which ROS production plays a central role, particularly during myocardial IR. Depending on concentration, ROS such as superoxide anion, hydrogen peroxide, and hydroxyl radicals may exert beneficial or deleterious effects on cells and tissues [68,69]. Under physiological conditions, low ROS levels function as redox signaling molecules, whereas excessive ROS production induces oxidative damage to macromolecules, promotes inflammation, and triggers cell death [70], primarily through mitochondrial dysfunction [71]. Superoxide anion is the predominant mitochondrial ROS and is rapidly converted to hydrogen peroxide by manganese superoxide dismutase (MnSOD) [72], an enzyme essential for aerobic life [73]. In the present study, MnSOD activity and protein levels in mitochondria were not affected by ISCH or IR. Unexpectedly, relatively high MnSOD levels were detected in the MAM fraction of control and ISCH hearts compared with IR hearts. To our knowledge, this is the first report demonstrating MnSOD localization within MAMs. Previous work has shown that mitochondrial matrix components can be exchanged through nanotunnels in response to Ca^2+^ imbalance [74]; thus, MnSOD presence in MAMs may exert a protective effect against impending mitochondrial ROS-mediated damage.

In cardiomyocytes, an initial slow increase in ROS is followed by a rapid burst known as ROS-induced ROS release [75]. This process is mediated by the inner mitochondrial anion channel [76] and is associated with transient ROS signaling events within MAMs. ROS generated by respiratory complexes also regulate Ca^2+^ transport between mitochondria and the SR. ROS localized within Ca^2+^ microdomains act as feedback regulators, enhancing further Ca^2+^ release via oxidation of SR RyR2 cysteine residues [77], albeit with the risk of excessive ROS accumulation and subsequent mitochondrial dysfunction.

Several antioxidant defense mechanisms maintain ROS at basal levels [78]. Under physiological conditions, hydrogen peroxide is detoxified by peroxiredoxin/thioredoxin systems, glutathione (GSH)-dependent enzymes, and GSH itself, which reflects cellular redox status. In 14-month-old hearts, glutathione peroxidase and glutathione reductase activities were preserved; however, thioredoxin reductase activity increased by 44.5% after ISCH, concomitant with reduced GSH levels. Notably, although ischemia reduced GSH levels to 64.2% of control, values returned to near baseline during reperfusion. This apparent normalization does not necessarily indicate attenuation of oxidative stress, as lipid peroxidation remained elevated. Instead, it likely reflects activation of compensatory antioxidant mechanisms. The observed increase in thioredoxin reductase activity suggests upregulation of NADPH-dependent redox systems, which may enhance glutathione recycling during early reperfusion. In addition, reperfusion is known to stimulate de novo GSH synthesis as part of an adaptive response to oxidative challenge [79]. Thus, the rebound in GSH levels likely represents a transient redox adaptation rather than full restoration of oxidative homeostasis. MAMs are enriched in proteins regulating redox metabolism [80], many of which are oxidoreductases capable of generating ROS and modulating SR Ca^2+^-handling proteins. Through multiorganellar redox complexes termed redoxosomes, these proteins influence SR–mitochondria Ca^2+^ flux and metabolic coupling [81] and regulate membrane contact site formation [80]. Moreover, MAMs have been implicated in the transmission of toxic lipid peroxidation products, such as lipid peroxides, to mitochondria [82]. In our study, myocardial ischemia and IR induced conjugated diene formation and structural alterations of membrane surfaces, evidenced by increased ANS probe binding to membrane proteins. Ischemia also promoted the formation of the lipid peroxidation product 4-hydroxy-2-nonenal (HNE), a reactive aldehyde capable of modifying proteins and altering MAM integrity, potentially impairing SR–mitochondria Ca^2+^ transfer or SR Ca^2+^ handling through ROS overproduction.

This study was performed only on male rats, so we cannot exclude the possibility that female rats may respond differently to ischemic injury due to hormonal effects. The experiments were conducted using isolated hearts in a Langendorff system, which allows precise control of ischemia–reperfusion but does not fully replicate the in vivo environment. Moreover, the study focused on acute ischemia–reperfusion with relatively short reperfusion times, which may not capture delayed recovery phenomena such as myocardial stunning. Future studies including female animals, in vivo models, and extended reperfusion periods are needed to address these limitations.

## 5. Conclusions

In summary, the present study demonstrates that myocardial ischemia and ischemia–reperfusion profoundly disrupt SR–mitochondria communication in middle-aged hearts, contributing to altered Ca^2+^ handling, redox imbalance, and impaired contractile recovery. Ischemia-induced remodeling of MAMs was characterized by reduced GRP75 and MFN2 levels, together with increased VDAC1 enrichment, suggesting dynamic reorganization of Ca^2+^-transfer contact sites in response to metabolic stress. These changes were associated with altered mitochondrial Ca^2+^ signaling, and dysregulated SR Ca^2+^ sequestration.

Although core mitochondrial antioxidant defenses such as MnSOD remained largely preserved, ischemia and reperfusion were associated with compartment-specific redox alterations at MAMs, as inferred from selective changes in redox enzyme activity and increased lipid peroxidation. This spatially restricted redox imbalance, occurring in parallel with remodeling of Ca^2+^-transfer machinery, likely enhances Ca^2+^-dependent ROS signaling generation at contact sites. Such localized Ca^2+^–ROS cross-talk may establish a feed-forward amplification loop that exacerbates mitochondrial dysfunction and contributes to cardiomyocyte injury.

Collectively, these findings identify SR–mitochondria coupling and MAM-associated redox regulation as critical determinants of cardiomyocyte resilience in the middle-aged heart. Although direct ROS measurements in isolated MAMs were not performed, our data support the concept that disruption of MAM integrity and Ca^2+^–redox cross-talk contributes to IR injury. Targeting MAM stability and localized redox homeostasis may therefore represent a promising strategy to improve cardiac outcomes in the elderly.

## Figures and Tables

**Table 1:** Effect of IR on contractile function parameters in hearts.

	CF (mL/min)	HR (bpm)	LV devP (mm Hg)	+LV dp/dT (mm Hg/s)	−LV dp/dT (mm Hg/s)
**CON**					
15 min	19.9 ± 0.2	185.7 ± 22.6	87.2 ± 3.1	1616.2 ± 129.2	1048.8 ± 104.9
65 min	20.3 ± 0.4	190.5 ± 15.7	88.9 ± 2.8	1635.6 ± 119.6	990.3 ± 130.6
**IR**					
before ISCH	20.1 ± 0.3	185.7 ± 22.6	87.6 ± 2.7	1566.7 ± 50.4	1004.8 ± 44.2
after IR	17.7 ± 0.1 ***	190.5 ± 15.7	69.5 ± 3.1 **	1026.2 ± 33.2 ***	751.8 ± 54.3 *

Values are expressed as mean ± SEM of 5 hearts per experimental group. A one-way ANOVA with post hoc comparisons by the Student–Neuman–Keuls test was carried out to test for significant differences (*) when compared to the preischemic value or control: * *p* < 0.05, ** *p* < 0.01, *** *p* < 0.001. CF (coronary flow), HR (heart rate), LV devP (left ventricular developed pressure), +LV dP/dT (maximum rate of pressure development), −LV dp/dT (maximum rate of relaxation).

**Table 2:** The effect of IR on antioxidant enzyme activities and glutathione content in hearts.

	CON	ISCH	IR
**MnSOD** U/mg protein	20.82 ± 0.97	22.35 ± 0.98	21.03 ± 1.10
**GR** μmol/(min.mg protein)	4.19 ± 0.31	3.96 ± 0.08	3.99 ± 0.07
**GPx** μmol/(min.mg protein)	5.21 ± 0.97	5.17 ± 0.25	5.39 ± 0.23
**TrxR** nmol/(min.mg protein)	38.31 ± 1.82	45.33 ± 1.35 *	40.2 ± 1.97
**GSH** μmol/g of tissue	1.62 ± 0.01	1.04 ± 0.03 ***	1.76 ± 0.07

Values are expressed as mean ± SEM of 5 hearts per experimental group. A one-way ANOVA with post hoc comparisons by the Student–Neuman–Keuls test was carried out to test for significant differences (*) when compared to the control: * *p* < 0.05, *** *p* < 0.001. MnSOD (Mn superoxide dismutase), GR (glutathione reductase), GPx (glutathione peroxidase), TrxR (thioredoxin reductase), GSH (reduced glutathione).

**Table 3:** The effect of IR on markers of lipid peroxidation and membrane hydrophobicity.

	CD A_233nm_/A_215nm_	HNE nmol/g Tissue	Lys-HNE ANS Probe Fluorescence Intensity (AU)	
**CON**	0.198 ± 0.008	12.57 ± 1.32	15.55 ± 1.76	110.83 ± 2.76
**ISCH**	0.336 ± 0.023 ***	21.82 ± 1.22 ***	16.23 ± 0.98	181.50 ± 4.04 ***
**IR**	0.287 ± 0.013 *	14.45 ± 1.18	15.73 ± 0.62	187.50 ± 3.59 ***

Values are expressed as mean ± SEM of 5 hearts per experimental group. A one-way ANOVA with post hoc comparisons by the Student–Neuman–Keuls test was carried out to test for significant differences (*) when compared to the control: * *p* < 0.05, *** *p* < 0.001. CD (conjugated dienes), HNE (4-hydroxy-2-nonenal), Lys-HNE (lysine conjugates with HNE), ANS (1-anilino-8-naphthalene sulfonate).

## Data Availability

All data related to this work can be made available upon request to the corresponding authors.

## Supplementary Materials

The following supporting information can be downloaded at: https://www.mdpi.com/article/10.3390/biomedicines14030547/s1, Figure S1: VDAC1 distribution in the purified mitochondrial fraction, crude mitochondrial and in the MAM fraction.

## Abbreviations

The following abbreviations are used in this manuscript:

ANS	1-anilino-8-naphthalenesulfonate
CD	Conjugated dienes
CF	Coronary flow
ER	Endoplasmic reticulum
GPx	Glutathione peroxidase
GR	Glutathione reductase
GRP75	Glucose-regulated protein 75
GSH	Reduced glutathione
HNE	4-hydroxy-2-nonenal
HR	Heart rate
IP3R	Inositol 1,4,5-trisphosphate receptor
LPO	Lipid peroxidation
LVP	Left ventricular pressure
LV devP	Left ventricular developed pressure
+LV dP/dt	Maximum rate of pressure development
−LV dP/dt	Maximum rate of relaxation
Lys-HNE	Lysine conjugates with HNE
MAMs	Mitochondria-associated membranes
MFN2	Mitofusin 2
MnSOD	Mn superoxide dismutase
ROS	Reactive oxygen species
RyR	Ryanodine receptor
SERCA	Sarco-endoplasmic reticulum Ca^2+^-ATPase
SR	Sarcoplasmic reticulum
TrxR	Thioredoxin reductase
VDAC1	Voltage-dependent anion channel 1

## Appendix A

The appendix table summarizes results of protein determination in tissue homogenates and isolated sarcoplasmic reticulum, mitochondria and mitochondria-associated membrane fractions.

**Table A1:** The effect of IR on body and heart weight, protein concentrations.

	Weight (g)		Protein Concentration (mg/mL of Protein)			
	Body	Heart	HOM	Pure MIT	SR	MAMs
**CON**	479.0 ± 24.2	1.6 ± 0.2	26.1 ± 1.4	4.0 ± 0.6	5.5 ± 0.7	1.2 ± 0.1
**ISCH**	482.8 ± 25.2	1.4 ± 0.2	18.7 ± 1.0 **	3.0 ± 1.0	5.1 ± 0.5	1.2 ± 0.2
**IR**	478.8 ± 6.2	1.6 ± 0.3	21.9 ± 1.5 *	3.7 ± 0.2	4.3 ± 0.6	1.1 ± 0.1

Values are expressed as mean ± SEM of 5 hearts per experimental group. A one-way ANOVA with post hoc comparisons by the Student–Neuman test was carried out to test for significant differences (*) when compared to the control: * *p* < 0.05, ** *p* < 0.01.
