# Supplementary material for: Disrupted SR–Mitochondria Coupling Drives Ischemia–Reperfusion Vulnerability in the Middle-Aged Rat Heart
Source: Biomedicines. 2026 Feb 27;14(3):547. doi: 10.3390/biomedicines14030547 (PMC13023512; doi:10.3390/biomedicines14030547)
Supplement: Supplementary file 1 [file biomedicines-14-00547-s001.zip › biomedicines-4158250-supplementary.pdf]

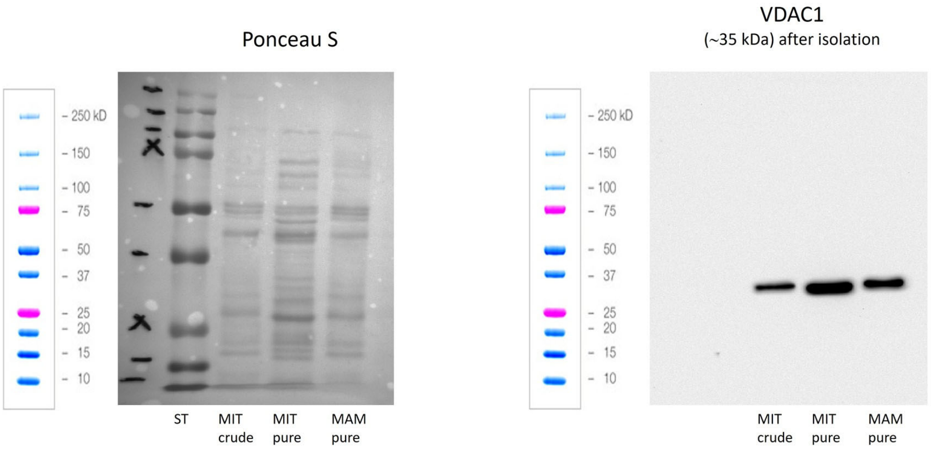

Figure S1. VDAC1 distribution in the purified mitochondrial fraction, crude mitochondrial and in the MAM fraction.
